# Printed and Flexible Capacitive Pressure Sensor with Carbon Nanotubes based Composite Dielectric Layer

**DOI:** 10.3390/mi10110715

**Published:** 2019-10-23

**Authors:** Zhenxin Guo, Lixin Mo, Yu Ding, Qingqing Zhang, Xiangyou Meng, Zhengtan Wu, Yinjie Chen, Meijuan Cao, Wei Wang, Luhai Li

**Affiliations:** Beijing Engineering Research Center of Printed Electronics, Beijing Institute of Graphic Communication, Beijing 102600, China; beiyinguozhenxin@163.com (Z.G.); 15366978028@163.com (Y.D.); zqq15201169516@163.com (Q.Z.); 17862328880@163.com (X.M.); wuzhengtanby@outlook.com (Z.W.); chenyinjie@bigc.edu.cn (Y.C.); caomeijuan@bigc.edu.cn (M.C.); wangwei@bigc.edu.cn (W.W.)

**Keywords:** capacitive pressure sensor, composite dielectric, printed and flexible sensor, carbon nanotubes, percolation theory

## Abstract

Flexible pressure sensors have attracted tremendous attention from researchers for their widely applications in tactile artificial intelligence, electric skin, disease diagnosis, and healthcare monitoring. Obtaining flexible pressure sensors with high sensitivity in a low cost and convenient way remains a huge challenge. In this paper, the composite dielectric layer based on the mixture of carbon nanotubes (CNTs) with different aspect ratios and polydimethylsiloxane (PDMS) was employed in flexible capacitive pressure sensor to increase its sensitivity. In addition, the screen printing instead of traditional etching based methods was used to prepare the electrodes array of the sensor. The results showed that the aspect ratio and weight fraction of the CNTs play an important role in improving the sensitivity of the printed capacitive pressure sensor. The prepared capacitive sensor with the CNTs/PDMS composite dielectric layer demonstrated a maximum sensitivity of 2.9 kPa^−1^ in the pressure range of 0–450 Pa, by using the CNTs with an aspect ratio of 1250–3750 and the weight fraction of 3.75%. The mechanism study revealed that the increase of the sensitivity of the pressure sensor should be attributed to the relative permittivity increase of the composite dielectric layer under pressure. Meanwhile, the printed 3 × 3 and 10 × 10 sensor arrays showed excellent spatial resolution and uniformity when they were applied to measure the pressure distribution. For further applications, the flexible pressure sensor was integrated on an adhesive bandage to detect the finger bending, as well as used to create Morse code by knocking the sensor to change their capacitance curves. The printed and flexible pressure sensor in this study might be a good candidate for the development of tactile artificial intelligence, intelligent medical diagnosis systems and wearable electronics.

## 1. Introduction

Recently, flexible pressure sensors have attracted widely attentions in the area of mobile bio-monitoring of disease diagnosis, tactile artificial intelligence, electric skin and healthcare services because of their real-time, convenient, and wearable features [1,2,3,4,5,6,7]. Among different types of flexible pressure sensors, the capacitive sensor owning the advantages of low energy consumption, fast response time, low detection limit and good sensing stability is the research hot topic in recent years [8,9]. Remarkable efforts have been made to improve the sensitivity of flexible capacitive sensors. According to the definition of the sensor’s sensitivity (σ) (Formula 1), it could be deduced that the sensitivity improvement could be mainly realized by two ways. Increase the distance change between two electrodes (d_0_/d) and the relative permittivity change of the dielectric layer (ε_r_/ε_r0_) under pressure. It should be noticed that the electrode area (A/A_0_) of the sensor is usually unchanged when the vertical pressure is applied.

(1) σ=(C−C0)C0P=d0d·εrεr0·AA0−1P

In Formula 1, P is the applied pressure, C_0_, d_0_, ε_r0_ and A_0_ are the capacitance, distance between electrodes, relative permittivity of the dielectric layer and the electrode area of the sensor without pressure respectively, C, d, ε_r_, and A are those parameters corresponding to the under pressure condition of sensor.

In the first route, the microstructures in electrodes and dielectric layers of the sensor were usually constructed to increase the electrodes distance change (d_0_/d) under pressure. For instance, Benjamin et al. [10] utilized the pyramidal microstructures in the dielectric layer to improve the sensitivity of the sensor and investigated the effects of the shape parameters and spacing on the sensor’s performance. Li et al. [11] used the bionic microstructures in the sensor electrodes which were duplicated from the lotus surface to obtain the sensitivity of 0.815 kPa^−1^ and fast response time of 38 ms. Lee et al. [12] reported the flexible capacitive sensor with porous dielectric layer of polydimethylsiloxane (PDMS), which showed a relatively high sensitivity of 1.18 kPa^−1^ in the low pressure range. However, such pressure sensors always exhibit complex fabrication process and have difficulties in controlling the uniformity of microstructures. In addition, the deformation of the microstructures tends to get saturation when the pressure increases, resulting in the high sensitivity of the flexile sensor could only remain in a relatively narrow and low pressure range, usually under 2 kPa [13,14].

On the other hand, the increase of the relative permittivity of the dielectric layer under pressure (ε_r_/ε_r0_) could also contribute to the improvement of the sensitivity (shown in Formula 1). However, this strategy is always ignored by researchers and lacks systemic study right now. Few works were reported that by adding the conductive filler into the polymer base to form composite dielectrics could increase their relative permittivity changes under pressure. For instance, Fang et al. [15] prepared the capacitive sensor by employing nickel-silicone rubber composite as the dielectric layer, which showed extreme high sensitivity of 460 kPa^−1^. Shi et al. [16] used the silver nanowires/PDMS composite as the dielectric layer of capacitive flexible sensor, which showed a sensitivity of 0.831 kPa^−1^ and a relatively low detect pressure of 1.4 Pa. Meanwhile, the carbon nanotubes (CNTs) were also used as the conductive filler to form the composite dielectrics. Jang et al. [14] revealed that the flexible sensor with alkylamine-functionalized multi-walled CNTs/PDMS composites dielectric layer demonstrated 1.8-fold increased capacitance change compared to its corresponding one with pure PDMS as a dielectric layer under the same pressure. Yoon et al. [17] prepared multifunctional capacitive sensor by also using CNTs/PDMS composite as dielectric layer, which were capable of pressure and temperature bimodal sensing performance. Although CNTs/polymer composites have been widely used in piezoresistive pressure sensor as the active layer [18,19,20], to our knowledge, their utilization as the dielectric layer in capacitive sensor is still limited. Furthermore, the mechanism of the sensitivity improvement by using the CNTs/polymer composite dielectric layer in the capacitive flexible sensor as well as the effects of the CNTs morphology on the relative permittivity change of the composite dielectric under pressure were not clearly yet.

In this paper, the flexible capacitive pressure sensor with CNTs/PDMS composite dielectric layer was prepared by screen printing method. The effects of the aspect ratios of the CNTs as well as their weight fraction and dispersion in the PDMS base to the relative permittivity change of composite dielectric were investigated. Furthermore, the sense mechanism of the prepared sensor was also studied to reveal the role of the composite dielectric played in the capacitive pressure sensor. Finally, the printed flexible pressure sensors were used to measure finger bending, pressure distribution and create the Morse code.

## 2. Materials and Methods

### 2.1. Materials

CNTs with different aspect ratios were purchased from Chengdu Institute of Organic Chemistry, Chinese Academy of Sciences (Chengdu, China). In this paper, we categorize the CNTs with their aspect ratios in the following discussion based on the reported literatures that the aspect ratio is the key factor influencing the dielectric property of the composite [21,22,23]. Their parameters are shown in Table 1. PDMS (SYLGARD 184) were purchased from Dow Corning (Midland, MI, USA). 1H,1H,2H,2H-Perfluorodecyltrichlorosilane as the surface modification agent to silicon wafer was purchased from TCI Shanghai Development Co., Ltd (Shanghai, China).

### 2.2. Preparation of theCNTs/PDMS Composites

**Figure 1:**
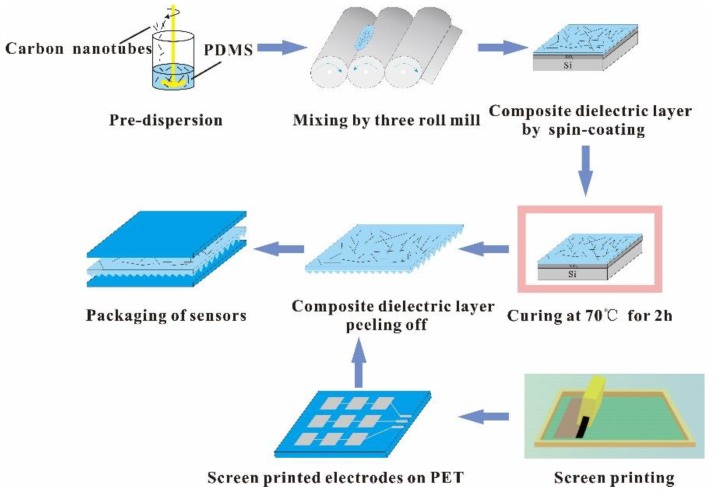
The schematic diagram for the fabrication of the flexible capacitive pressure sensor.

The CNTs/PDMS composites with 1, 2, 3, 3.75 wt.% of CNTs (AR: 1250–3750) and 1, 2, 3, 4, 5, 6 wt.% of CNTs (AR: 500–3000 and 200–600) were prepared respectively, as shown in Figure 1. The different CNTs weight fractions in the composites are mainly attributed to the different dispersion performances of CNTs fillers with different aspects ratios. The CNTs with the relatively high aspect ratio of 1250–3750, the highest mass fraction which makes the composites homogeneous and stable is 3.75 wt.%. For the CNTs with the aspect ratios of 500–3000 and 200–600, the composites remain their stability and homogeneity in the highest mass fraction of 6 wt.%. The CNTs with calculated weight fraction, PDMS elastomer and the curing agent with the weight ratio of 10:1 were mechanically stirred for pre-dispersion. Then, the composites were further uniformly dispersed by a three-roll mill machine (EXAKT80E, EXAKT Technologies, Inc. Norderstedt, Germany) for 10 min to obtain well dispersed and stable CNTs/PDMS composite dielectrics. The mill speed was 400 r/min and the roller gap was 5 μm.

### 2.3. Preparation of the Capacitive Flexible Pressure Sensors

Pyramid-shaped microstructures of CNTs/PDMS dielectric layer was employed to improve the sensitivity of the flexible sensor. First, the silicon wafer with the pyramidal microstructure, having a base of 40 μm, height of 28 μm and the periodic spacing of 140 μm, was prepared using photolithography technology. Then the CNTs/PDMS composites with different weight fractions and aspect ratios of CNTs were spin coated on the silane-treated silicon mold at 2000 rpm following by curing at 70 °C for 2 h. After that, the CNTs/PDMS composites with the microstructures were peeled off from the silicon wafer. On the other sides, the electrode arrays were screen printed on the polyethylene terephthalate (PET) substrates using the nano-silver ink which was prepared according to our previously published ref [24]. In this paper, two kinds of electrode arrays, 3 × 3 and 10 × 10 with the sensing pixel area of 1 cm^2^ and 4 cm^2^, were prepared by screen printing (OS-500FB, Ou Laite Printing Machinery Industry Co., Ltd., Jiangsu, China). Finally, the CNTs/PDMS composite dielectric layer was packaged between two printed electrodes with sandwich structure to integrate a capacitive pressure sensor. As a comparison, the pressure sensor using pristine PDMS as dielectric layer was also prepared in the same method descripted above. The prepared capacitive sensor was used to measure the pressure distribution, detect the finger bending and create Morse code as the applied demonstration.

### 2.4. Characterization

**Figure 2:**
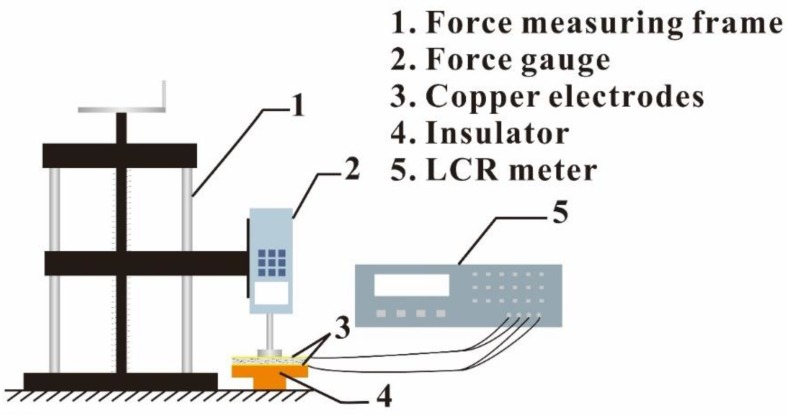
The schematic diagram for the piezo-capacitive effect experimental setup.

Dispersion and morphology of the CNTs fillers in PDMS bases were observed in cross section view by scanning electron microscope (SEM, SU8020, HITACHI, Tokyo, Japan). The dielectric properties of the CNTs/PDMS composites were measured using the network analyzer at the frequency of 120 MHz (Agilent E4990A, Agilent Technologies Inc., Minneapolis, Hennepin, MI, USA). To measure the relative permittivity of the composites film under pressure, the samples were compressed with the pressure of 35 kPa for 48 h, following by the measurement immediately after releasing the pressure using a LCR meter (TH2617, Changzhou Tonghui Electronics Co., Ltd., Jiangsu, China). The schematic for the capacitive sensor experimental setup is shown in Figure 2. Capacitances of the CNTs/PDMS composites under different pressure were measured using the TH2617 LCR meter in air under the condition of 1 V at 10 kHz.

## 3. Results and Discussion

### 3.1. Dielectric Property of CNTs/PDMS Composite

**Figure 3:**
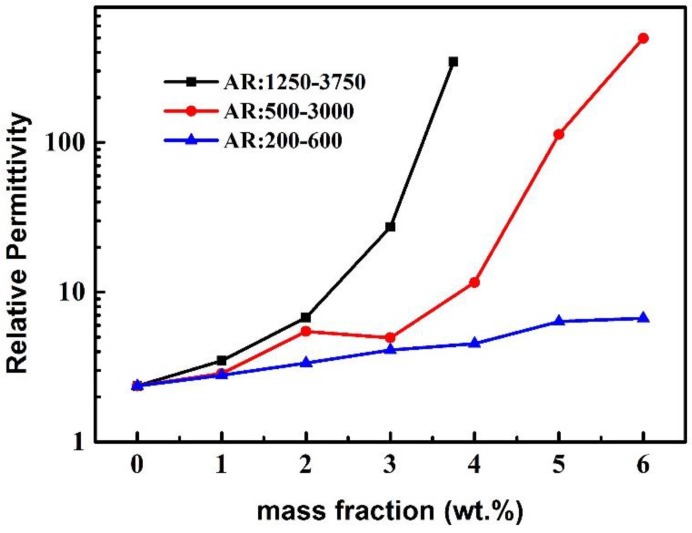
The relative permittivities of the CNTs/ PDMS composites versus the CNTs mass fractions.

The CNTs mass fraction related relative permittivities of the CNTs/PDMS composites at the frequency of 1.2 MHz were measured shown in Figure 3. For all the CNTs fillers with different aspect ratios, the relative permittivities of the CNTs/PDMS composite dielectrics increase as the mass fraction of CNTs increasing. For instance, the relative permittivity of the composite dielectric using CNTs fillers with the aspect ratio of 200–600 slightly increases to 6.6 when the mass fraction of CNTs reaches at 6 wt.%, which is 2.75 times to that of pure PDMS (shown in Table 2). While for both the CNTs fillers with the aspect ratios of 500–3000 and 1250–3750, their mass fraction related relative permittivity curves show an obvious increase when the CNTs mass fractions reach a certain values. For the CNTs filler with the aspect ratio of 500–3000, the relative permittivity of the composite sharply increases at the CNTs mass fraction of 3 wt.%. When the CNTs mass fraction increases from 3 wt.% to 6 wt.%, the relative permittivity of the composite goes from 4.95 to 497, which is a 100-fold increase. For CNTs filler with the aspect ratio of 1250–3750, the drastically increase of the relative permittivity of the composite begins at the CNTs mass fraction of 2 wt.% and sharply increases to 3.75 wt.%. And their corresponding relative permittivities are 6.77 and 323.4 respectively, which are 2.82 and 134.7 times increased compared to those of pure PDMS. It should be noticed that when the mass fraction of the CNTs with the aspect ratio of 1250–3750 exceeds to 3.75 wt.%, the CNTs cannot be dispersed well in the PDMS base, thus it is not discussed in this paper.

The results in Figure 3 and Table 2 show that the relative permittivity of the CNTs/PDMS composite is strongly depended on the aspect ratio and mass fraction of the CNTs fillers. The relative high aspect ratio of the CNTs contributes to faster increase in the relative permittivity of composite compared to that of relatively low aspect ratio CNTs. This phenomenon could be explained by the percolation theory, which is used to predict the electrical properties of a percolation system with non-interacting randomly dispersed fillers. The value of the relative permittivity ε_r_ is obtained as Formula 2 in this research [25], where fc is the percolation weight fraction of the CNTs filler, f_CNTs_ is the actual weight fraction of CNTs, ε_rPDMS_ is the relative permittivity of PDMS and S is an exponent constant. According to Formula 2, the relative permittivity of the composite dielectric (ε_r_) increases as the CNTs filler weight fraction (f_CNTs_) increasing. Meanwhile, the growth rate of the relative permittivity speeds up when the CNTs mass fraction (f_CNTs_) approaches the percolation threshold (f_c_) of the CNTs/PDMS composites. It is clear that the tendency of the mass dependent relative permittivity curves of the CNTs/PDMS composites in Figure 3 accords with the percolation theory well.

(2) $$\varepsilon_{r}\propto \varepsilon_{r\;\mathrm{PDMS}}(f_{c}-f_{\mathrm{CNTs}}){}^{-S},\;f_{c}\geq f_{\mathrm{CNTs}}$$

**Figure 4:**
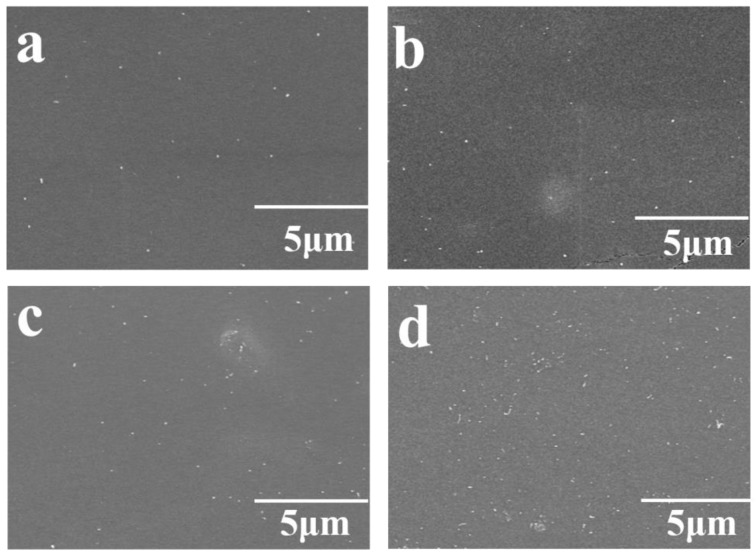
The cross-sectional views of CNTs/PDMS composite films based on different mass fraction of CNTs with the aspect ratios of 1250–3750, (**a**) 1 wt.%, (**b**) 2 wt.%, (**c**) 3 wt.%, (**d**) 3.75 wt.%.

To further investigate the morphology and dispersion of the CNTs in PDMS base, the cross-sectional views of the CNTs/PDMS composites using CNTs with aspect ratio of 1250–3750 as filler in different mass fractions were observed by SEM (Figure 4). As shown in Figure 4, most of the CNTs are in the horizontal direction along the composite film, resulting in the ends of the CNTs are viewed in the cross section. It should be attributed to the centrifugal force of the spin coating process to prepare the CNTs/PDMS composite film. The CNTs are dispersed in PDMS base with homogeneous and their distribution density in the cross section gradually increases as their mass fraction increasing, which accounts for the increase in ε_r_ shown in Figure 3. On the other hand, the interfacial polarization is another possible reason for the increase of the composite dielectrics’ relative permittivity [26]. The increase of the interfaces between CNTs filler and the PDMS base as the mass fraction of CNTs increasing could further enhance the electron mobility and interface polarization, resulting in the improvement of the relative permittivity [27].

### 3.2. CNTs/PDMS Composite Under Pressure

**Figure 5:**
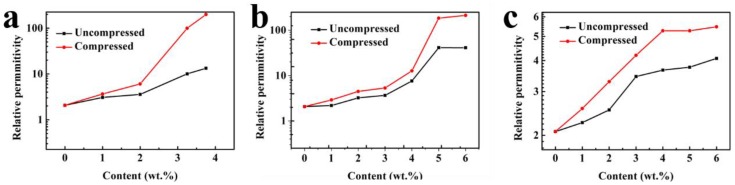
Mass fraction related relative permittivity of the composites based on CNTs filler with different aspect ratios with and without pressure, (**a**) 1250–3750, (**b**) 500–3000, (**c**) 200–600.

To simulate the situation of the sensor application, the dielectric property of the CNTs/PDMS composite under pressure was also investigated. Figure 5 shows the mass fraction related relative permittivities of the CNTs/PDMS composites based on different aspect ratio CNTs filler with and without pressure. All the curves in Figure 5 related to the CNTs/PDMS composites under pressure rise up compared to those of without pressure. In addition, the gaps between the two curves increase as the CNTs mass fraction increases. The results in Figure 5 indicate that the CNTs filler has a positive role in increase the relative permittivity of the dielectric layer of capacitive pressure sensor under pressure (ε_r_/ε_r0_), which is beneficial to the improvement of the sensitivity shown in Formula 1. Specifically, as shown in Figure 5a and Table 3, the relative permittivity of CNTs/PDMS composite based on the CNTs filler with aspect ratio of 1250–3750 at the mass fraction of 3.75 wt.% increases from 13.3 without pressure to 198.92 under pressure, which is 14.95 times enhanced. For the CNTs fillers with the aspect ratios of 500–3000 and 200–600 respectively (Figure 5b,c), the maximum relative permittivity enhancements of their corresponding composites are both at the CNTs mass fraction of 6 wt.%. And their relative permittivities under pressure increased 5.17 and 1.09 times respectively compared to those of without pressure (shown in Table 3). The relative permittivity enhancement of the CNTs/PDMS composites under pressure should be mainly attributed to two reasons. First, the CNTs/PDMS composite film is compressed when pressure is applied. The compressed composite gives rise to a concentration increase of the CNTs fillers in the vertical direction. As confirmed in Section 3.1, the relative permittivity of the composite increases as the concentration of CNTs fillers increasing. Second, the electric field between the two electrodes at a given potential difference is also strengthened under pressure because the electrodes distance is reduced as the composite film compressed. Therefore, the corresponding interfacial polarization between CNTs fillers and PDMS base is also reinforced, resulting in the relative permittivity enhancement under pressure [27].

### 3.3. Effect of the CNTs/PDMS Composite Dielectric on the Performance of Capacitive Flexible Pressure Sensor

**Figure 6:**
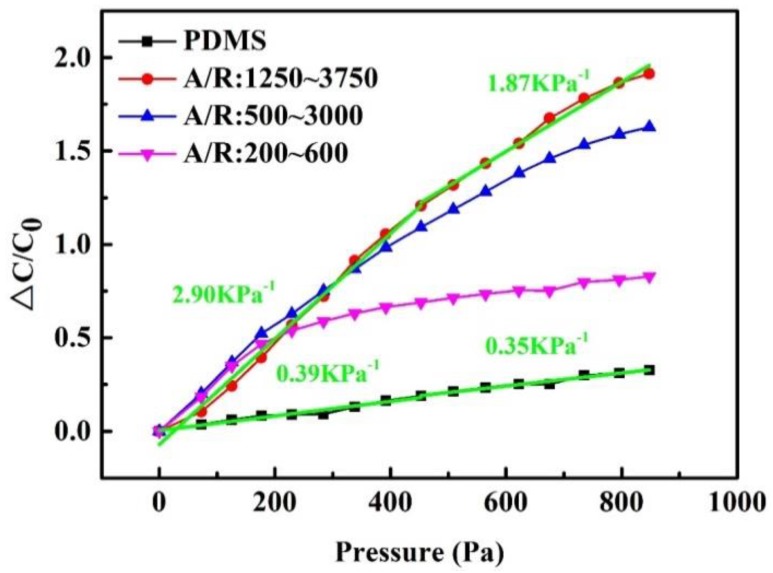
Capacitance change of the flexible sensors with composite dielectric layer based on various aspect ratios of CNTs under pressure.

Figure 6 shows change in capacitance ΔC/C_0_ versus applied pressure for the flexible sensors with CNTs/PDMS composite dielectric layer. The slopes of the curves which are numerically equal to the sensitivity of the pressure sensors are calculated. The sensitivities of the flexible sensors based on composite dielectric layers are obviously higher than that of based on pure PDMS as the dielectric layer. This is consistent with the former results discussed in Section 3.1 and Section 3.2. The CNTs/PDMS composite dielectric shows larger relative permittivity change than that of pure PDMS under pressure, which gives rise to the relatively higher sensitivity. Especially, the flexible sensor using CNTs of the aspect ratio 1250–3750 as filler shows the sensitivity of 2.90 kPa^−1^ in the pressure range of 0–450 Pa and 1.87 kPa^−1^ in the range of 450–850 Pa, which are 7.4 times and 5.3 times higher compared to that of pure PDMS based sensor respectively. The performance of the flexible sensor in this work, including the sensitivity and the linear range, is superior to most of reported capacitive pressure sensors using the similar composites as summarized in Table 4. Thus the CNTs/PDMS composite using CNTs with the aspect ratio of 1250–3750 and weight fraction of 3.5 wt.% as the fillers is chosen as the dielectric layer of the printed sensors to demonstrate their applications in the following.

### 3.4. Applications of the Printed and Flexible Pressure Sensor

**Figure 7:**
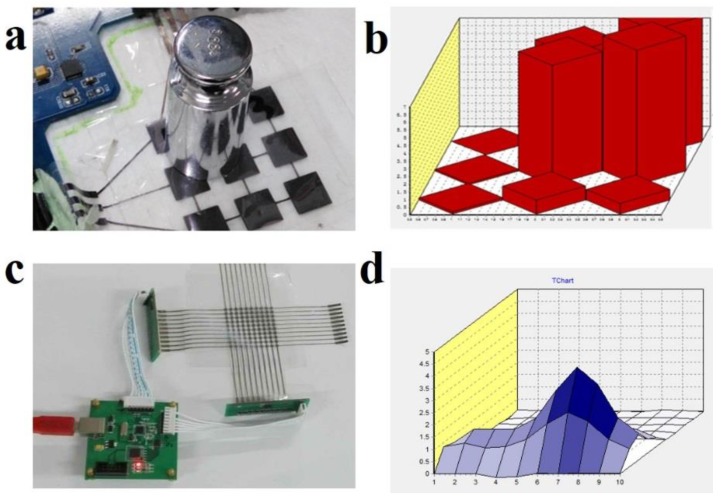
(**a**) The printed flexible 3 × 3 capacitive pressure sensor array and (**b**) its measurement of the 100 g weight pressure distribution; (**c**) The 10 × 10 pressure sensor array and (**d**) its corresponding testing pressure distribution map.

The flexible capacitive pressure sensor array was prepared to demonstrate their applications with screen printed electrodes and CNTs/PDMS composite dielectric layers. As shown in Figure 7a, a 3 × 3 printed sensor array was employed to measure the pressure distribution of a 100 g weight. The capacitance responses of four weight located sensor pixels are homogeneous (Figure 7b) which means that the printed 3 × 3 sensor array shows relatively good uniformity. To further improve the capacity of printed sensor measuring the pressure distribution with higher resolution, the 10 × 10 printed and flexible pressure sensor array was prepared shown in Figure 7c. Each sensor pixel is 1 cm × 1 cm area. The testing pressure distribution map in Figure 7d shows that the capacitance change gradually reduces from the center to the surrounding smoothly.

**Figure 8:**
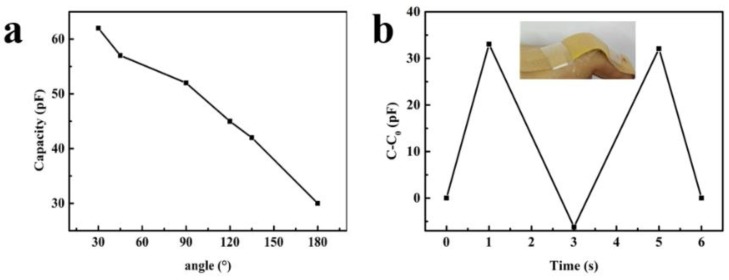
(**a**) The bending angle related capacitance of flexible sensor; (**b**) The capacitance change C-C_0_ response to repeating finger bending. Inset is the flexible pressure sensor integrated on a bandage to detect the finger bending.

Another application of the prepared flexible capacitive sensor was to measure the finger bending movement by utilizing the characteristics of the sensor that its capacitance changes with the different bending angles. As shown in Figure 8a, the capacitance of the flexible sensor decreases from 62 pF to 30 pF as the bending angle from 30° to 180° with a relatively good linear characteristic. Figure 8b shows the capacitance change C-C_0_ of the repeating finger bending movement.

**Figure 9:**
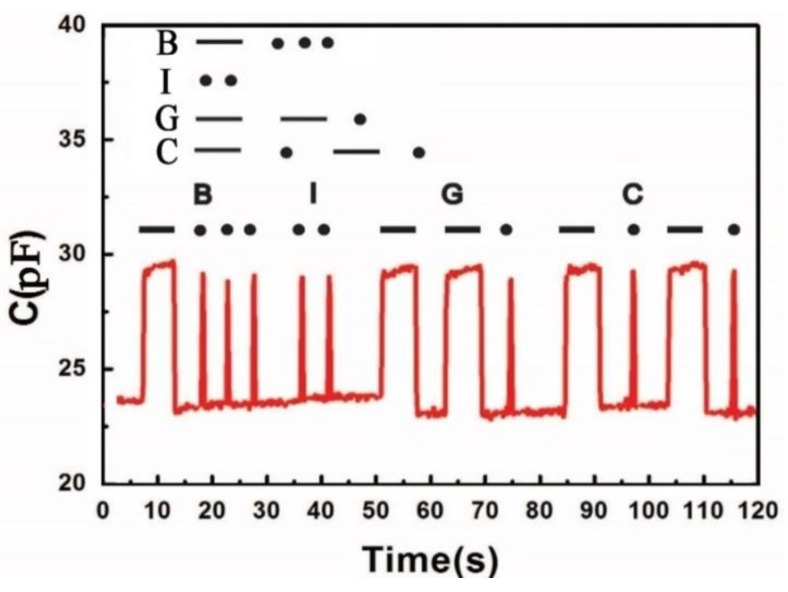
Time dependent capacitance of the prepared flexible sensor by knocking the sensor with finger to create Morse code. The word of “BIGC” which means Beijing Institute of Graphic Communication was yielded by converting the capacitance curve wave into Morse code.

Finally, the prepared flexible pressure sensor was used as a demonstrator to create Mores code. As shown in Figure 9, the time depended capacitance curve is formed to different “dots” and “dashes” by knocking the sensor with finger, which correspond to the letters of “BIGC” in Mores code. The sharp cut edge of the capacitance curve indicates that the prepared flexible sensor has an ultra-fast response time and relatively high stability.

## 4. Conclusions

In conclusion, the CNTs/PDMS composite dielectric layer was prepared to improve the sensitivity of printed and flexible pressure sensor. The results showed that the CNTs filler with a relatively high aspect ratio in certain degree could be a better candidate for the flexible capacitive pressure sensor compared to the corresponding CNTs with low aspect ratio. For composite film based on 3.75 wt.% CNTs with the aspect ratio of 1250–3750, the relative permittivity increases to 198.92 under pressure of 35 kPa, which is 14.95 times enhanced to that of without pressure. This is a significant contribution to increase the sensitivity of the flexible capacitive sensor. In results, the pressure sensor using pyramid-structural CNTs /PDMS composite dielectric layer possesses sensitivity of 2.90 kPa^−1^ in the range of 0–450 Pa and 1.87 kPa^−1^ in the range of 450–850Pa. The printed 3 × 3 and 10 × 10 capacitive pressure sensor array were successfully prepared and applied to measure pressure distribution. Meanwhile, the printed flexible sensors were also used to detect the finger bending and create Morse code. This research demonstrates that the sensors with microstructural CNTs/PDMS composite dielectric layer have great potential applications for healthcare monitoring, e-skins and human–computer interaction.

## Figures and Tables

**Table 1:** The parameters of the carbon nanotubes (CNTs) conductive fillers.

Physical Parameters	TNMH1	TNMH3	TNMH7
Diameter (nm)	<8	10–20	30–50
Purity (wt.%)	>98	>98	>98
Length (μm)	10–30	10–30	10–20
Aspect ratio	1250–3750	500–3000	200–600

**Table 2:** The relative permittivities of the CNTs/ PDMS composites with different aspect ratios CNTs as filler at certain mass fraction.

Composites with Different Aspect Ratio CNTs	Pure PDMS	AR:1250–3750		AR:500–3000		AR:200–600	
		2wt.%	3.75wt.%	3wt.%	6wt.%	3wt.%	6wt.%
Relative permittivity	2.4	6.77	323.4	4.95	497	4.10	6.6
Increased times to pure PDMS	/	2.82	134.7	2.06	207	1.7	2.75

**Table 3:** The relative permittivities of the CNTs/PDMS composites with different aspect ratios CNTs fillers with and without pressure.

Composites with Varied Aspect Ratios CNTs	without Pressure	with Pressure	Increase
Pure PDMS	2.07	2.07	/
AR: 1250–3750 (3.75 wt.%)	13.3	198.92	×14.95
AR: 500–3000 (6 wt.%)	41.06	212.52	×5.17
AR: 200–600 (6 wt.%)	4.08	5.47	×1.09

**Table 4:** The main characteristics of various capacitive-type pressure sensors.

Sensing Membrane Material	Maximum Sensitivity (kPa^−1^)	Linear Range (kPa)	Ref.
Porous PDMS	1.18	0–0.2	[12]
Porous Ecoflex	2.306	0–0.06	[28]
Nano-needle structured PDMS	1.76	0–0.2	[29]
Silver nanowires/PDMS pyramids	0.831	0–0.3	[16]
Zinc oxide nanowires/poly(methyl methacrylate) composites	0.095	0–0.1	[30]
This work	2.90	0–0.45	/

## Abbreviations

CNTs	Carbon nanotubes
PDMS	Polydimethylsiloxane
PET	Polyethylene terephthalate
